# Acute Toxin-mediated Rhabdomyolysis During Treatment With Trimethoprim-sulfamethoxazole

**DOI:** 10.5811/cpcem.2019.7.42688

**Published:** 2019-09-23

**Authors:** Michael Sperandeo, Dorjan Pantic, Jessica Army

**Affiliations:** * Northwell Health at North Shore University Hospital, Department of Emergency Medicine, Manhasset, New York; † Northwell Health at Long Island Jewish Medical Center, Department of Emergency Medicine, New Hyde Park, New York; ‡ Zucker School of Medicine at Hofstra/Northwell, Hempstead, New York

## Abstract

Rhabdomyolysis is a condition in which skeletal muscle breakdown causes the release of intracellular components into the bloodstream – defined as elevations in serum creatine kinase levels. The etiology of rhabdomyolysis is varied and may be the result of toxin-mediated mechanisms or metabolic derangements, or they may develop secondary to other conditions such as seizures, trauma and prolonged immobilization. In this case, we present a patient with suspected acute toxin-mediated rhabdomyolysis in the setting of trimethoprim-sulfamethoxazole (TMP-SMX) therapy for urinary tract infection. To our knowledge, this marks the fifth case report of an otherwise healthy patient diagnosed with rhabdomyolysis thought to be secondary to TMP-SMX.

## INTRODUCTION

While not an uncommon diagnosis in the emergency department, rhabdomyolysis is often difficult to diagnose clinically and may present in a variety of contexts and clinical scenarios. Patients often present with non-specific generalized myalgias, aches, fatigue, urine color changes, and/or fevers. Others present without symptoms. Rhabdomyolysis presents on a spectrum from asymptomatic elevations in serum creatine kinase (CK) to life-threatening acute electrolyte disturbances requiring hemodialysis.^1^–^2^

Identification of rhabdomyolysis usually begins in the proper clinical context based on history and physical, a suspected mechanism, and elevations in CK detected on laboratory analysis. Although an elevation of CK levels at least five-fold greater than the upper limit of normal is a commonly accepted parameter for definitive diagnosis, no true consensus exists.^3^–^4^ The etiology of rhabdomyolysis is varied; it may be the result of pharmacologic or biologic toxin-mediated mechanisms, or metabolic derangements, or it may develop secondary to other conditions such as seizures, trauma, and prolonged immobilization.^1^–^3^ When considering the precipitating mechanism, it is helpful to consider four main categories: ischemic, pharmacologic, physical, and biologic.^1^ Commonly cited medications with a known association with rhabdomyolysis include statins and fibrates. Recreational drugs, psychiatric mediations, and a variety of antimicrobial agents have also been associated with acute rhabdomyolysis.^1^,^2^,^5^

In this case, we present a patient with suspected acute toxin-mediated rhabdomyolysis in the setting of new trimethoprim-sulfamethoxazole (TMP-SMX) therapy for urinary tract infection (UTI). While rhabdomyolysis in the setting of TMP-SMX has been well described in the immunocompromised, no widespread association exists with immunocompetent patients. To our knowledge, this marks the fifth case report of an otherwise healthy, immunocompetent patient diagnosed with rhabdomyolysis thought secondary to TMP-SMX with no other clear insult or mechanism to explain elevations in CK levels.

## CASE REPORT

A 41-year-old African American male with a past medical history of overactive bladder, benign prostatic hyperplasia, obstructive sleep apnea requiring nocturnal home continuous positive airway pressure, presented to the emergency department (ED) with a chief complaint of increasing proximal bilateral lower extremity muscle pain for five days. Pain was associated with increasing difficulty with ambulation. The patient also noted persisting hematuria, urgency and hesitancy for the prior nine days. He had fever to 101.2° Fahrenheit (F) for the prior five days. Symptoms were associated with intermittent episodes of nausea and vomiting for the prior two days. Four days prior to presentation, the patient reported having seen his outpatient urologist who diagnosed a UTI. He was prescribed TMP-SMX double strength 160 milligrams (mg)/800mg. and endorsed medication compliance.

On ED arrival, the patient was found to be febrile to 102.3°F and hemodynamically stable. On exam, he appeared grossly uncomfortable. The bilateral lower extremity proximal muscle strength was 4/5 without point tenderness. There was no evidence of lower extremity edema, erythema or rash. Distal pulses were intact. The patient was also noted to have mild left lower quadrant abdominal and suprapubic tenderness to palpation; there was no costovertebral angle tenderness to palpation. Physical exam was otherwise unremarkable.

Initial laboratory analysis showed an aspartate transaminase (AST) 274 units per liter (u/L) (reference range, 4–40 u/L), alanine transaminase (ALT) 102 u/L (reference range, 4–41 u/L), CK muscle/brain 3.11 nanograms per milliliter (ng/mL) (reference range, 1–6.6 ng/mL) with a CK 60,665 ng/mL (reference range, 30–200 u/L). Labs obtained from the patient’s outpatient urologist from four days prior to presentation showed an initial leukocytosis to 14.4 10^3^ per cubic millimeter (mm^3^), which resolved prior to ED arrival, a urine analysis consistent with UTI: positive large blood with greater than 50 red blood cells per high-powered field, 50 white blood cells per high-powered field, and positive leukocyte esterase. A urine culture was positive for *Escherichia coli* sensitive to TMP-SMX per the outpatient urologist. The outpatient provider did not request CK levels at initial visit, but at that time AST/ALT levels were 22 u/L and 30 u/L, respectively.

Chest radiograph and computed tomography of the abdomen and pelvis in the ED were negative for acute pulmonary or intra-abdominal pathologies. In the ED, the patient received one gram of ceftriaxone for UTI, two liters of normal saline, acetaminophen for fever, and morphine for pain control. TMP-SMX was discontinued. He was admitted to the inpatient internal medicine service with a concern for non-traumatic acute rhabdomyolysis and pyelonephritis. Further workup by the inpatient medicine team excluded other possible causes for acute rhabdomyolysis. Respiratory viral panel and human immunodeficiency virus studies were negative. Urine toxicology was positive for opiates. Epstein-Barr viral studies were consistent with a prior infection with immunity. Thyroid and glucose-6-phosphate dehydrogenase studies were normal. CK and AST levels peaked on days two and three of admission, respectively (Figure).

The patient continued to receive intravenous and oral fluids as well as pain control. Over the eight days of admission the patient continued to improve. He reported decreasing muscle pain and was increasingly able to ambulate without difficulty. During the course of his hospital stay, the patient sustained no electrolyte abnormalities, and creatinine and blood urea nitrogen were within normal limits. There was no evidence of acute kidney injury. The patient was discharged on day eight with resolution of symptoms and a down-trending of CK and AST/ALT.

**CPC-EM Capsule**

What do we already know about this clinical entity?

*Rhabdomyolysis may develop secondary to metabolic derangements, seizures, trauma, or prolonged immobilization, or via a toxin-mediated mechanism*.

What makes this presentation of disease reportable?

*We present a case of trimethoprim-sulfamethoxazole (TMP-SMX) induced rhabdomyolysis in an otherwise healthy patient*.

What is the major learning point?

*In otherwise unclear etiologies of rhabdomyolysis in applicable patients, recent TMP-SMX use should be considered as a possible precipitating insult*.

How might this improve emergency medicine practice?

*Early recognition of this possible emerging association between TMP-SMX and rhabdomyolysis in immunocompetent patients may alter emergency department management and treatment*.

## DISCUSSION

Trimethoprim-sulfamethoxazole is a bacterial folic acid synthesis inhibitor used to treat a variety of conditions including UTI, skin and soft tissue infections in healthy patients, and pneumocystis pneumonia or toxoplasmosis in the immunocompromised patient. Side effects generally are mild and include rash, fever, muscle aches and diarrhea. TMP-SMX is also known to have an association with more severe side effects including Steven-Johnson syndrome and toxic epidermal necrolysis, although these are quite rare.^6^ TMP-SMX induced rhabdomyolysis is more often seen in the setting of immunocompromised patients or in the setting of allogeneic stem cell transplant recipients.^7^–^8^ To our knowledge, this report now represents the fifth case of TMP-SMX induced rhabdomyolysis in the setting of an otherwise healthy, immunocompetent patient.

In 2014 Ainapurapu et al. reported a case of a previously healthy 40-year-old Hispanic female. She initially presented with two days of bilateral lower extremity muscle aches and weakness. She reported she was prescribed TMP-SMX for a UTI two days prior and had taken four doses. She presented with an initial CK of 20063 u/L, with an otherwise negative workup for other etiologies explaining her rhabdomyolysis.^8^

A second case in 2015 by Petrov et al. was that of a 64-year-old male, active smoker, who also presented complaining of bilateral lower extremity pain and a decreasing ability to ambulate secondary to discomfort. The patient reported self-treating a presumed UTI with TMP-SMX left over from a prior episode of UTI. He reported taking TMP-SMX 40mg/800mg three times a day for two weeks. On first presentation to the ED, the patient was found to have a CK of 1524 u/L, and he was referred to a neurologist with suspicion for polymyositis. TMP-SMX was not discontinued and the patient’s pain continued to worsen. The patient also was prescribed ibuprofen, celecoxib, piroxicam, ketorolac, and meloxicam. On subsequent presentations to the ED, the patient was found to have a CK of 614,691 u/L, elevated transaminases, and multiple electrolyte derangements. Muscle biopsy was suggestive of muscle fibers without striations and a loss of homogeneity. Other causes of rhabdomyolysis were ruled out. TMP-SMX was discontinued, and over the course of admission the patient reported improvement in all symptomatology and was subsequently discharged.^9^

In 2017, Moye et al. presented a case of a 43-year-old African American female with a past medical history of depression and alcohol-use disorder who presented to the ED after eight days of bilateral lower extremity leg pain in the setting of taking five doses of TMP-SMX 160mg/800mg for a UTI. She reported a history of daily alcohol consumption and 10mg vortioxetine. She first began to experience leg pain two days after initiation of TMP-SMX when she re-presented to her primary care provider. There she was switched to ciprofloxacin. The patient continued to experience worsening leg pain, which prompted her visit to the ED. There she was found to have a CK of 26,231 u/L with elevated transaminases AST/ALT of 352/111 u/L, respectively.^10^ She was admitted, started on intravenous (IV) fluids and folic acid supplementation. During her admission, CK peaked at 45,020 u/L without increase in serum creatinine. She was discharged on day eight after improvement in her lower extremity discomfort and with a CK of 2809 u/L.

Also in 2017, Goyal et al. presented a case of an 18-year-old female with a past medical history of Arnold-Chiari malformation presenting from her primary care provider (PCP) complaining of severe weakness and diffuse muscle pains in all extremities for two days. She endorsed recent completion of a course of TMP-SMX for UTI from her PCP. At time of admission, she was noted to have severe proximal muscle weakness in all four extremities and diffuse muscle tenderness. On admission her CK level was 20,418 u/L, which peaked the next day to greater than 42,670 u/L, the maximal quantifiable limit per the institution’s laboratory. She initially received aggressive fluid resuscitation without improvement. Ultimately, she was started on IV immunoglobulin G with eventual resolution of elevations in CK.

There are a few concerning similarities with each of these presentations. In each case TMP-SMX was initiated for a presumed UTI. Subsequently, each patient presented complaining of bilateral lower extremity proximal muscle weakness or pain. Each case demonstrated elevations in CK consistent with rhabdomyolysis. TMP-SMX was discontinued resulting in improvement of clinical status and resolution of CK levels. Each patient had a negative workup for other etiologies of rhabdomyolysis and was without concern for immunocompromised state.

There are certainly possible confounding factors in each patient presentation. Confounders include but are not limited to pre-existing medical conditions and past medical history, outpatient prescription medications, home self-medication, substance abuse and non-modifiable factors including gender and age. However, the overarching consistencies – indication for therapy, the temporal relationship to initiation of TMP-SMX therapy, and chief complaint at presentation – are noteworthy and are fairly consistent between cases.

## CONCLUSION

After evaluating for and eliminating other possible toxic, biologic or physical and traumatic etiologies of acute rhabdomyolysis in our patient, we concluded that TMP-SMX was the most likely insulting agent. Furthermore, while serum CK was not obtained prior to initiation of TMP-SMX, the temporal relationship of normal AST four days prior to presentation – and parallel trending of AST/ALT with CK at time of presentation and during admission– suggests that our patient’s rising AST/ALT was likely secondary to skeletal muscle breakdown and may serve as a surrogate for CK. TMP-SMX was discontinued and over the course of admission the CK, proximal lower extremity myalgia, ability to ambulate, and overall clinical status gradually improved.

In each of the four prior cases, as with ours, the indication for TMP-SMX therapy was a UTI. Our case report adds to a growing body of anecdotal evidence that suggests TMP-SMX may be associated with acute rhabdomyolysis in immunocompetent patients. While the authors are not aware of any large, multicenter study evaluating for a true association of TMP-SMX with rhabdomyolysis, additional larger scale studies are warranted. Providers should be made aware of this possible emerging association between TMP-SMX and rhabdomyolysis in immunocompetent patients.

## Figures and Tables

**Figure:**
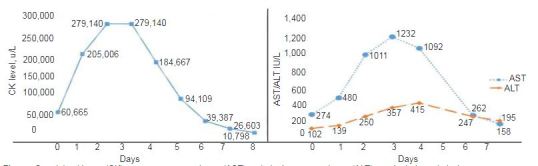
Creatinine kinase (CK), aspartate transaminase (AST) and alanine transaminase (ALT) trends during admission.
